# Low levels of influenza H5N1 HA and NA antibodies in the human population are boosted by seasonal H1N1 infection but not by H3N2 infection or influenza vaccination

**DOI:** 10.1128/mbio.02145-25

**Published:** 2025-10-31

**Authors:** Anne P. Werner, Cosette G. Schneider, Elgin H. Akin, Juliahna Hayes, Katherine Z. J. Fenstermacher, Richard E. Rothman, Lynda Coughlan, Andrew Pekosz

**Affiliations:** 1 W. Harry Feinstone Department of Molecular Microbiology and Immunology, The Johns Hopkins Bloomberg School of Public Health, Baltimore, Maryland, USA; 2 Department of Microbiology and Immunology, University of Maryland School of Medicine, Baltimore, Maryland, USA; 3 Department of Emergency Medicine, Johns Hopkins University School of Medicine, Baltimore, Maryland, USA; 4 Center for Vaccine Development and Global Health (CVD), University of Maryland School of Medicine, Baltimore, Maryland, USA; Boston University Chobanian & Avedisian School of Medicine, Boston, Massachusetts, USA

**Keywords:** H5N1, neuraminidase, hemagglutinin, population immunity, neutralizing antibody, neuraminidase inhibiting antibody

## Abstract

An increase in the number of human cases of influenza A/H5N1 infection in the USA has raised concerns about the pandemic potential of the virus. Pre-existing population immunity is a key determinant for risk assessment and pandemic potential for any virus. Antibody responses against the bovine A/H5N1 hemagglutinin (HA) and neuraminidase (NA) proteins were measured among a population of influenza-vaccinated or influenza-infected individuals. Modest titers of bovine A/H5N1 HA-binding antibodies and low to undetectable neutralizing antibody titers were detected in a cohort of 73 individuals. Conversely, bovine A/H5N1 NA-binding and neuraminidase-inhibiting antibody titers were comparable to those against a human A/H1N1 NA at baseline. Seasonal influenza vaccination failed to significantly increase antibody titers against both HA and NA glycoproteins of bovine A/H5N1. Recent infection with human A/H1N1 but not A/H3N2 viruses induced significant increases in bovine A/H5N1-neutralizing antibody, as well as increases in NA-binding and NA-inhibiting antibodies to bovine A/H5N1 NA. While the degree of protection afforded by these A/H5N1 cross-reactive antibodies is not known, incorporating NA or enhancing current seasonal vaccine formulations to increase NA-specific antibody titers may increase antibody breadth and protection against both seasonal and pandemic influenza viruses.

**IMPORTANCE:**

A/H5N1 influenza A viruses continue to pose a pandemic threat to humans. Recent infection of dairy cattle and poultry with A/H5N1 in the USA has magnified that concern. We determined the level of antibodies that recognize A/H5N1 hemagglutinin (HA) and neuraminidase (NA) proteins in a population in Baltimore, MD. We show that while low levels of H5 HA-binding and A/H5N1-neutralizing antibodies are present, there is a significantly stronger recognition of bovine N1 NA. Vaccines that target the N1 NA protein may induce protective antibody responses in humans due to the presence of cross-reactive human N1 NA antibodies.

## INTRODUCTION

Since its arrival in North America in late 2021, highly pathogenic avian influenza (HPAI) A/H5N1 clade 2.3.4.4b has been a major threat to human and animal health (1–3). Despite only sporadic outbreaks in humans, with nearly all cases directly traceable to exposures to infected animals, and the lack of evidence of human-to-human transmission, the high case fatality rate of roughly 52% seen globally for related H5N1 clades substantiates significant concern over its pandemic potential (1, 3, 4).

The A/H5N1 virus has spread to a large number of migratory birds, and that has led to hundreds of spillovers into a range of wild mammals, avian species such as birds of prey (not usually hosts for avian influenza viruses), domesticated poultry, household cats, and dairy cows. The A/H5N1 clade 2.3.4.4b viruses have also undergone reassortment with other avian influenza viruses, leading to a number of different genotypes, including B3.13 (responsible for most dairy cow infections) and D1.1 (driving a large number of poultry farm infections) (5).

As of June 2025, the US Centers for Disease Control report 70 cases of A/H5N1 infection, primarily in individuals working in dairy or poultry farms with known exposures to infected animals (2). While one death has been reported, the vast majority of cases have resulted in relatively mild infection, often limited to conjunctivitis (6–8). This is in contrast to both outbreaks of A/H5N1 HPAI in prior years and contemporary outbreaks outside of North America (9–11). For example, of the 10 reported cases of A/H5N1 in Cambodia in 2025 alone, 6 of them have been fatal (11, 12). Sporadic spillovers have occurred to humans with no evidence of human-to-human transmission (1, 3, 4). A/H5N1 viruses isolated from a subset of these infections have shown amino acid changes consistent with adaptation to replication in mammals, which highlights concerns about the virus evolving into a form that may lead to a pandemic (13–15).

Because A/H5NX (where X is one of a number of neuraminidase subtypes) influenza viruses have not circulated in the human population, the vast majority of humans lack detectable antibodies against the H5 hemagglutinin (HA) protein—the major antigenic target of the influenza immune response. However, human seasonal A/H1N1 viruses encode a neuraminidase (NA) protein that is antigenically and structurally similar to that of A/H5N1 viruses. Experiments in animals and surveys of human cohorts have indicated that antibodies to seasonal human A/H1N1 NA can cross-react with the A/H5N1 NA, but it is unknown if these low-level cross-reactive antibodies can provide protection against or mediate severity of A/H5N1 HPAI infection in humans (16–19).

To determine the level of pre-existing human population immunity to bovine-derived A/H5N1 viruses, serum samples from two distinct cohorts—healthcare workers vaccinated with the 2024 seasonal influenza vaccine and individuals infected with seasonal A/H1N1 or A/H3N2 influenza during the 2023–2024 influenza season—were tested for the presence of antibodies recognizing the H5 HA or N1 NA proteins. Functional antibody responses—neutralizing antibody (nAb) and neuraminidase-inhibiting (NAI) antibody—against bovine A/H5N1 HA and NA were quantified in both cohorts. Low levels of binding IgG to the bovine H5 HA correlate with minimal-to-undetectable nAb titers to bovine A/H5N1 viruses, and neither binding IgG titers nor nAb are boosted by standard seasonal vaccination. Similarly, binding IgG and NAI antibody titers to human seasonal A/H1N1 NA and bovine A/H5N1 NA were quantified and were comparable at baseline (i.e., prior to vaccination or infection). Increases in NA-specific antibody to both human and bovine N1 NAs following infection with seasonal A/H1N1 viruses, but not A/H3N2 viruses, were detected. Lastly, despite no detectable change in N1-binding titers post-A/H3N2 infection, baseline serum depletion of A/H3N2 NA-specific antibody reduces total bovine N1-binding IgG, suggesting that heterotypic NA antibody may play a role in baseline cross-reactivity. Our findings corroborate and extend existing evidence that current seasonal vaccine formulations are poor at inducing NA-specific humoral responses (18, 20).

## RESULTS

### Study design and demographics

Population-level antibody responses to A/H5N1 HPAI NA in healthy adults were investigated utilizing a cohort of individuals employed by the Johns Hopkins Hospital (JHH) or Johns Hopkins Medical Institutes (JHMI) who were receiving their annual dose of trivalent 2024–2025 Northern Hemisphere (NH) seasonal inactivated influenza vaccine in September and October 2024. Vaccinees provided blood samples at the time of enrollment, indicated as “day 0,” or “baseline,” and at day 28 post-vaccination (Fig. S1a). Fifty participants were selected, comprised of 25 males and 25 females, with ages ranging between 23 years and 68 years (Table 1). Forty-seven of 50 (94%) participants received egg-grown vaccine (Fluarix, GSK), and the remaining participants (6%) were immunized with standard trivalent cell-grown vaccine (Flucelvax, Seqirus) (Table 1). A minor subset (4%) of all participants reported that they had not received any influenza vaccination in any of the past five NH influenza seasons (Table 1). Of the remaining (96%) participants, the average number of previous vaccinated seasons was 4.46/5.

**TABLE 1:** Vaccine cohort characteristics, demographics, and geometric mean titers (GMTs) for all assessments described^*a*^

	By sex				By age group				
	Female	Male	Total	*P*-value	18–44	45–55	56–70	Total	*P*-value
*n* (%)	25 (50.00)	25 (50.00)	50 (100.00)		24 (48.00)	12 (24.00)	14 (28.00)	50 (100.00)	
Female, *n* (%)	25 (100.00)	0 (0.00)	25 (50.00)		10 (41.67)	9 (75.00)	6 (42.86)	25 (50.00)	
Age in years, mean (SD)	44.44 (13.86)	44.44 (14.12)	44.44 (13.85)	1.00	31.71 (6.40)	50.25 (2.73)	61.29 (2.84)	44.44 (13.85)	
BMI, mean (SD)	27.52 (5.75)	27.88 (5.61)	27.70 (5.63)	0.82	25.88 (5.36)	28.36 (4.30)	30.36 (6.26)	27.70 (5.63)	0.06
Race, *n* (**%**)									
White	19 (76.00)	14 (56.00)	33 (66.00)	0.23	15 (62.50)	9 (75.00)	9 (64.29)	33 (66.00)	0.80
Hispanic/Latino	<4	<4	5 (15.15)	>0.99	<4	<4	0 (0.00)	5 (15.15)	0.48
Non-hispanic/Non-latino	16 (84.21)	12 (85.71)	28 (84.85)	>0.99	12 (80.00)	7 (78.78)	9 (100.00)	28 (84.85)	0.48
Black	<4	7 (28.00)	10 (20.00)	0.29	5 (20.83)	<4	4 (35.71)	10 (20.00)	0.53
Hispanic/Latino	0 (0.00)	0 (0.00)	0 (0.00)		0 (0.00)	0 (0.00)	0 (0.00)	0 (0.00)	
Non-Hispanic/Non-Latino	<4	7 (100.00)	10 (100.00)		5 (100.00)	<4	4 (100.00)	10 (100.00)	
Asian/Pacific Islander	<4	<4	<4	>0.99	<4	<4	0 (0.00)	<4	0.60
American Indian or Alaska Native	0 (0.00)	0 (0.00)	0 (0.00)		0 (0.00)	0 (0.00)	0 (0.00)	0 (0.00)	
Other	<4	<4	4 (8.00)	>0.99	<4	<4	<4	4 (8.00)	>0.99
N/A	0 (0.00)	0 (0.00)	0 (0.00)		0 (0.00)	0 (0.00)	0 (0.00)	0 (0.00)	
Comorbidities, *n* (**%**)									
Transplant recipient	0 (0.00)	0 (0.00)	0 (0.00)		0 (0.00)	0 (0.00)	0 (0.00)	0 (0.00)	
Cancer (ongoing or remission)	<4	0 (0.00)	<4	0.49	0 (0.00)	<4	<4	<4	0.27
Type II diabetes	<4	5 (20.00)	6 (12.00)	0.19	0 (0.00)	<4	5 (35.71)	6 (12.00)	**0.004**
Autoimmune disease	0 (0.00)	0 (0.00)	0 (0.00)		0 (0.00)	0 (0.00)	0 (0.00)	0 (0.00)	
Immunosuppressive medication	0 (0.00)	0 (0.00)	0 (0.00)		0 (0.00)	0 (0.00)	0 (0.00)	0 (0.00)	
Methotrexate			0 (0.00)					0 (0.00)	
Tacrolimus			0 (0.00)					0 (0.00)	
Mycophenolate			0 (0.00)					0 (0.00)	
Other			0 (0.00)					0 (0.00)	
Hepatic disease	0 (0.00)	0 (0.00)	0 (0.00)		0 (0.00)	0 (0.00)	0 (0.00)	0 (0.00)	
Renal disease	<4	0 (0.00)	0 (0.00)	>0.99	<4	0 (0.00)	0 (0.00)	<4	>0.99
Cardiovascular disease	<4	<4	4 (8.00)	0.61	0 (0.00)	<4	<4	4 (8.00)	0.088
Chronic lung disease	<4	<4	4 (8.00)	0.61	<4	<4	0 (0.00)	4 (8.00)	0.25
Asthma	<4	0 (0.00)	<4	>0.99	<4	<4	0 (0.00)	<4	>0.99
Other	0 (0.00)	<4	<4	>0.99	<4	0 (0.00)	0 (0.00)	<4	>0.99
Neurological disorder	0 (0.00)	0 (0.00)	0 (0.00)		0 (0.00)	0 (0.00)	0 (0.00)	0 (0.00)	
Epilepsy			0 (0.00)					0 (0.00)	
Stroke			0 (0.00)					0 (0.00)	
Hematological disease	<4	0 (0.00)	<4	0.49	<4	0 (0.00)	0 (0.00)	<4	>0.99
Sickle cell disease	0 (0.00)		0 (0.00)		0 (0.00)			0 (0.00)	
Anemia (non-sickle cell)	<4		<4		<4			<4	
Other	<4		<4		<4			<4	
Reproductive disease	7 (28.00)	0 (0.00)	7 (14.00)	**0.0003**	<4	5 (41.67)	0 (0.00)	7 (14.00)	0.09
PCOS	<4		<4		<4	0 (0.00)		<4	
Endometriosis	0 (0.00)		0 (0.00)		0 (0.00)	0 (0.00)		0 (0.00)	
Primary ovarian insufficiency	0 (0.00)		0 (0.00)		0 (0.00)	0 (0.00)		0 (0.00)	
Hysterectomy (full or partial)	4 (57.14)		4 (57.14)	**0.02**	0 (0.00)	4 (80.00)		4 (57.14)	**0.0046**
Other	<4		<4	>0.99	<4	<4		<4	>0.99
Endocrine/Metabolic disease	<4	<4	4 (8.00)	0.35	<4	<4	<4	4 (8.00)	0.42
Thyroid	<4	<4	4 (100.00)	0.61	<4	<4	<4	4 (100.00)	0.68
Smoking history	<4	<4	5 (10.00)	>0.99	<4	<4	<4	5 (10.00)	0.84
Current smoker?	<4	<4	<4	>0.99	0 (0.00)	<4	<4	<4	0.58
<4	<4	<4	<4	>0.99	<4	0 (0.00)	<4	<4	0.58
Vaccine history**,** *n* (**%**)					<4				
Seasonal vaccine NH 2024–2025	25 (100.00)	25 (100.00)	50 (100.00)	>0.99	24 (100.00)	12 (100.00)	14 (100.00)	50 (100.00)	>0.99
No seasonal vaccine NH 2023–2024	<4	<4	4 (8.00)	0.61	<4	<4	0 (0.00)	4 (8.00)	0.45
No seasonal vaccine in any of previous five seasons	0 (0.00)	<4	<4	0.09	<4	0 (0.00)	0 (0.00)	<4	0.28
Baseline immunity, GMT (geom. SD)					<4				
Bovine A/H5N1 HA IgG	367.31 (2.74)	519.06 (3.46)	436.64 (3.11)	>0.99	262.63 (2.30)	364.88 (3.16)	1217.39 (2.54)	436.64 (3.11)	**0.0004**
A/H1N1 nAb (NT_50_*^d^* titer)	139.29 (2.88)	169.12 (1.93)	153.48 (2.38)	0.55	219.83 (2.32)	95.14 (2.20)	124.91 (2.12)	153.48 (2.38)	0.05
Bovine A/H5N1-LAIV^*b*^ nAb (NT_50_ titer)	7.58 (1.82)	10.57 (1.69)	8.95 (1.97)	0.15	7.94 (2.01)	10.00 (2.06)	10.00 (1.84)	8.95 (1.97)	0.59
A/H1N1 Cal09 NA IgG	2,930.24 (5.58)	3,043.97 (4.22)	2,986.56 (4.81)	>0.99	4,387.06 (5.60)	1,366.43 (4.82)	3,019.65 (2.86)	2,986.56 (4.81)	0.77
Bovine A/H5N1 NA IgG	1,475.32 (4.47)	1,500.18 (4.82)	1,487.70 (4.57)	>0.99	1,807.56 (5.49)	1,177.61 (5.02)	1,302.40 (3.04)	1,487.70 (4.57)	>0.99
A/H1N1 ELLA^*c*^ NAI_50_ titer	1,381.02 (3.14)	1,501.34 (3.28)	1,439.92 (2.79)	>0.99	11,992.20 (3.30)	1,048.04 (2.32)	1,083.65 (2.86)	1,439.92 (2.79)	0.18
Bovine A/H5N1-LAIV ELLA NAI_50_ titer	151.49 (2.51)	205.75 (2.38)	176.55 (2.46)	0.37	195.39 (2.59)	135.52 (2.44)	182.12 (2.29)	176.55 (2.46)	0.88
Post-vaccination immunity, GMT (geom. SD)									
Bovine A/H5N1 HA IgG	480.17 (2.65)	667.89 (2.81)	566.30 (2.74)	>0.99	352.07 (2.11)	561.80 (2.65)	1,287.89 (2.58)	566.30 (2.74)	**0.0007**
A/H1N1 nAb (NT_50_ titer)	311.25 (3.07)	263.55 (3.29)	286.41 (2.59)	>0.99	339.03 (2.55)	239.73 (3.32)	249.83 (2.12)	286.41 (2.59)	0.64
Bovine A/H5N1-LAIV nAb (NT_50_ titer)	9.46 (1.88)	12.48 (2.16)	10.87 (1.86)	0.20	9.44 (1.84)	10.00 (2.19)	14.86 (1.43)	10.87 (1.86)	0.12
A/H1N1 Cal09 NA IgG	3,221.64 (6.16)	3,465.78 (4.07)	3,341.48 (4.99)	>0.99	5,317.07 (5.98)	1,648.72 (4.54)	2,761.12 (3.06)	3,341.48 (4.99)	0.67
Bovine A/H5N1 NA IgG	1,513.06 (4.63)	1,506.60 (5.20)	1,509.83 (4.83)	>0.99	1,775.16 (5.88)	1,203.63 (5.60)	1,389.21 (2.98)	1,509.83 (4.83)	>0.99
A/H1N1 ELLA NAI_50_ titer	1,964.50 (2.66)	2,462.03 (3.19)	2,199.24 (2.36)	0.29	2,879.32 (2.04)	1,886.27 (3.07)	1,580.57 (2.07)	2,199.24 (2.36)	0.05
Bovine A/H5N1-LAIV ELLA NAI_50_ titer	187.07 (2.25)	272.28 (2.30)	225.69 (2.30)	0.19	241.45 (2.44)	181.37 (2.47)	242.45 (1.97)	225.69 (2.30)	>0.99

^*a*^ For qualitative data and comorbidities, Fisher’s exact test was used to generate *P*-values shown. For baseline and post-vaccination immunity readouts, adjusted *P*-values shown were calculated via Kruskal-Wallis nonparametric test with Bonferroni’s correction for age analyses, and Dunn’s test for multiple comparisons for sex analyses, both with Bonferroni’s corrections. Bolded *P*-values are significant (i.e., *P* < 0.05). Percentages are calculated as total of parent category. For demographic characteristics in which the *n* was below four persons, the value is indicated as “<4”.

^*b*^ LAIV, live attenuated influenza vaccine.

^*c*^ ELLA, enzyme-linked lectin assay.

^*d*^ NT₅₀, neutralizing titer 50%.

To investigate the role of seasonal IAV infection in shaping cross-reactive antibody responses to bovine A/H5N1 HA and NA, a cohort of patients who presented to the JHH Emergency Department or were inpatients at the JHH with influenza-like illness (ILI) with the confirmed seasonal IAV infection during the 2023–2024 Northern Hemisphere influenza season were utilized. IAV infection was confirmed via point-of-care diagnostic tests or next-generation sequencing. All 23 patients provided blood samples at the time of admittance, hereafter referred to as “baseline,” and again at approximately 4 weeks later, hereafter referred to as “convalescent” (Fig. S1b). Sixteen of 23 (69.6%) patients were infected with A/H1N1pdm09-like viruses, and 7 (30.4%) with A/H3N2-like viruses (Table 2). Fourteen of 23 (60.9%) participants were female, and 9 of 23 (39.1%) were male (Table 2). Fewer than four patients (13%) were solid organ transplant recipients and on immunosuppressive medication (Table 2). Fewer than four patients (8.7%) had previously undergone chemotherapeutic cancer treatment and were in remission at the time of the study (Table 2). Thirteen of 23 (56.7%) patients had reported receipt of a seasonal influenza vaccine for the 2023–2024 NH season (Table 2). For comorbidities, five patients (21.7%) reported cardiovascular disease, five patients (21.7%) reported hematological disorders, fewer than four patients (8.7%) reported renal disease, and fewer than four patients (13%) reported asthma (Table 2).

**TABLE 2:** Infection cohort characteristics, demographics, and geometric mean titers (GMTs) for all assessments described^*c*^

	By IAV subtype responsible for infection				By sex			
	A/H1N1pdm09	A/H3N2	Total	*P*-value	Female	Male	Total	*P*-value
*n* (%)	16 (69.57)	7 (30.43)	23 (100)		14 (60.87)	9 (39.13)	23 (100)	
Female, *n* (%)	12 (75.0)	2 (28.57)	14 (60.87)	0.07				
A/H1N1pdm09-infected, *n* (%)					12 (85.71)	4 (44.44)	16 (69.57)	0.07
Age in years, mean (SD)	48.25 (14.54)	53.57 (17.76)	49.87 (15.38)	0.94	48.79 (17.27)	51.56 (12.66)	49.87 (15.38)	0.86
BMI, mean (SD)	29.12 (6.18)	31.57 (10.04)	29.90 (7.46)	1.00	30.39 (7.64)	29.19 (7.59)	29.90 (7.46)	1.00
Race, *n* (%)								
White	0 (0.00)	0 (0.00)	0 (0.00)		0 (0.00)	0 (0.00)	0 (0.00)	
Hispanic/Latino								
Non-Hispanic/Non-Latino								
Black	16 (100.00)	6 (85.71)	22 (95.65)	0.30	13 (92.86)	9 (100.00)	22 (95.65)	>0.99
Hispanic/Latino	<4	<4	<4	0.21	<4	<4	<4	>0.99
Non-Hispanic/Non-Latino	15 (93.75)	5 (83.33)	20 (90.91)	0.21	12 (85.71)	8 (88.89)	20 (90.91)	>0.99
Asian/Pacific Islander	0 (0.00)	<4	<4	0.30	<4	0 (0.00)	<4	>0.99
American Indian or Alaska Native	0 (0.00)	0 (0.00)	0 (0.00)		0 (0.00)	0 (0.00)	0 (0.00)	
Other	0 (0.00)	0 (0.00)	0 (0.00)		0 (0.00)	0 (0.00)	0 (0.00)	
N/A								
Comorbidities, *n* (%)								
Transplant recipient	<4	<4	<4	>0.99	<4	<4	<4	>0.99
Cancer (ongoing or remission)	<4	0 (0.00)	<4	0.53	<4	0 (0.00)	<4	>0.99
Type II diabetes	4 (25.00)	<4	5 (21.74)	>0.99	<4	<4	5 (21.74)	0.26
Autoimmune disease	<4	0 (0.00)	<4		<4	0 (0.00)	1 (4.35)	
Immunosuppressive medication	<4	0 (0.00)	<4	0.53	<4	<4	3 (13.04)	>0.99
Methotrexate	0 (0.00)	0 (0.00)	0 (0.00)		0 (0.00)	0 (0.00)	0 (0.00)	
Tacrolimus	<4	0 (0.00)	<4		0 (0.00)	<4	1 (4.35)	
Mycophenolate	<4	0 (0.00)	<4		<4	0 (0.00)	1 (4.35)	
Other	<4	0 (0.00)	<4		<4	0 (0.00)	1 (4.35)	
Hepatic disease	<4	<4	<4	0.30	0 (0.00)	<4	0 (0.00)	0.04
Renal disease	<4	0 (0.00)	<4	>0.99	<4	<4	<4	>0.99
Cardiovascular disease	4 (25.00)	<4	5 (21.74)	0.62	<4	<4	<4	0.34
Chronic lung disease (asthma)	<4	<4	<4	0.53	<4	0 (0.00)	<4	0.25
Neurological disorder	<4	<4	<4	>0.99	<4	0 (0.00)	<4	0.25
Epilepsy	<4	0 (0.00)	<4		<4	0 (0.00)	<4	
Stroke	<4	<4	<4		<4	0 (0.00)	<4	
Hematological disease	5 (31.25)	0 (0.00)	5 (21.74)	0.27	5 (35.71)	0 (0.00)	5 (21.74)	0.12
Sickle cell disease	<4	0 (0.00)	<4	0.41	<4	0 (0.00)	<4	0.18
Anemia (non-sickle cell)	<4	0 (0.00)	<4	0.41	<4	0 (0.00)	<4	0.18
Other	<4	0 (0.00)	<4		<4	0 (0.00)	<4	
Reproductive disease	<4	0 (0.00)	<4	>0.99	<4	0 (0.00)	<4	0.49
PCOS	<4	0 (0.00)	<4		<4	0 (0.00)	<4	
Other	<4	0 (0.00)	<4		<4	0 (0.00)	<4	
Endocrine/Metabolic disease (other than diabetes)	<4	0 (0.00)	<4		<4	0 (0.00)	<4	
Thyroid	<4	0 (0.00)	<4		<4	0 (0.00)	<4	
Smoking history	9 (56.25)	<4	12 (52.17)	0.67	8 (57.14)	4 (44.44)	12 (52.17)	0.68
Current smoker?	5 (55.56)	<4	8 (66.67)	>0.99	6 (75.00)	<4	8 (66.67)	0.62
Past smoker?	4 (44.44)	0 (0.00)	4 (33.33)	>0.99	<4	<4	4 (33.33)	0.62
Vaccine/Infection history, *n* (**%**)								
Receipt of seasonal vaccine NH 2023–2024	9 (56.25)	4 (57.12)	13 (56.65)	0.37	8 (57.14)	5 (55.55)	13 (56.65)	>0.99
No seasonal vaccine in any of the previous five seasons	4 (25.00)	<4	6 (26.08)	0.62	<4	<4	6 (26.08)	0.34
Known exposure to confirmed influenza positive case	<4	<4	4 (17.39)	>0.99	4 (28.57)	0 (0.00)	4 (17.39)	0.34
Baseline immunity, GMT (geom. SD)								
Bovine A/H5N1 HA IgG	272.23 (2.56)	197.71 (3.58)	246.98 (2.81)	>0.99	283.41 (3.17)	199.39 (2.29)	246.98 (2.81)	0.62
A/H1N1 nAb (NT_50_*^f^* titer)	28.28 (3.46)	20.00 (2.45)	25.45 (3.12)	>0.99	31.23 (3.64)	18.52 (2.24)	25.45 (3.12)	0.74
Bovine A/H5N1-LAIV^*d*^ nAb (NT_50_ titer)	8.05 (1.63)	10.00 (2.23)	8.60 (1.80)	>0.99	9.52 (1.89)	7.35 (1.65)	8.60 (1.80)	0.65
A/H1N1 Cal09 NA IgG	1,010.74 (4.94)	1,497.94 (3.17)	1,139.30 (4.31)	>0.99	917.81 (4.86)	1,594.70 (3.54)	1,139.30 (4.31)	0.94
Bovine A/H5N1 NA IgG	1,487.97 (4.42)	8,250.42 (10.57)	2,509.76 (6.80)	0.37	2,405.46 (3.08)	3,450.07 (16.51)	2,509.76 (6.80)	>0.99
A/H1N1 ELLA^*e*^ NAI_50_ titer	2,742.37 (10.97)	1,401.21 (5.04)^*a*^	2,283.46 (8.97)^*a*^	>0.99	2,884.27 (12.03)	1,517.28 (5.08)^*a*^	2,283.46 (8.97)^*a*^	>0.99
Bovine A/H5N1-LAIV ELLA NAI_50_ titer	84.43 (2.03)	134.15 (1.63)	97.21 (1.96)	0.25	103.41 (2.04)	88.29 (1.90)	97.21 (1.96)	>0.99
Post-infection immunity, GMT (geom. SD)								
Bovine A/H5N1 HA IgG	1,726.92 (4.94)	405.14 (2.37)	1,110.79 (4.72)	0.18	1,529.86 (6.10)	675.12 (2.51)	1,110.79 (4.72)	>0.99
A/H1N1 nAb (NT_50_ titer)	246.75 (7.68)	16.41 (3.04)	108.14 (8.94)	**0.004**	168.12 (12.35)	54.43 (4.19)	108.14 (8.94)	0.71
Bovine A/H5N1-LAIV nAb (NT_50_ titer)	15.42 (2.03)	9.06 (2.10)	13.12 (2.11)	0.14	18.11 (2.14)	7.94 (1.41)	13.12 (2.11)	**0.013**
A/H1N1 Cal09 NA IgG	44,767.45 (7.52)	1,481.40 (2.21)	15,864.97 (10.47)	**0.003**	23,768.27 (9.26)	8,459.58 (12.53)	15,864.97 (10.47)	>0.99
Bovine A/H5N1 NA IgG	32,608.38 (7.37)	5,399.50 (3.32)	18,864.32 (7.08)	0.058	29,065.89 (5.6)	9,629.35 (2.51)	18,864.32 (7.08)	0.69
A/H1N1 ELLA NAI_50_ titer	31,520.78 (9.39)	803.53 (4.08)^*b*^	13,157.77 (13.37)^*b*^	**0.008**	27,516.16 (12.83)^*a*^	3,967.56 (10.2)^*a*^	13,157.77 (13.37)^*a*^	0.16
Bovine A/H5N1-LAIV ELLA NAI_50_ titer	524.67 (3.78)	168.90 (2.38)	371.59 (3.67)	0.12	544.46 (3.89)	205.11 (2.73)	371.59 (3.67)	0.26

^*a*^ One sample of the indicated group was not run due to limited sample volume.

^*b*^ Two samples of the indicated group were not run due to limited sample volume. For demographic characteristics in which the *n* was below four persons, the value is indicated as “<4”.

^*c*^ For qualitative data and comorbidities, Fisher’s exact test was used to generate *P*-values shown. For baseline and post-vaccination immunity readouts, adjusted *P*-values shown were calculated via Dunn’s test for multiple comparisons with Bonferroni’s correction. Bolded *P*-values are significant (i.e., *P* < 0.05). Percentages are calculated as total of parent category. Percentages are calculated as total of parent category.

^*d*^ LAIV, live attenuated influenza vaccine.

^*e*^ ELLA, enzyme-linked lectin assay.

^*f*^ NT₅₀, neutralizing titer 50%.

### Baseline cross-reactive antibodies to bovine A/H5N1 NA

**Fig 1:**
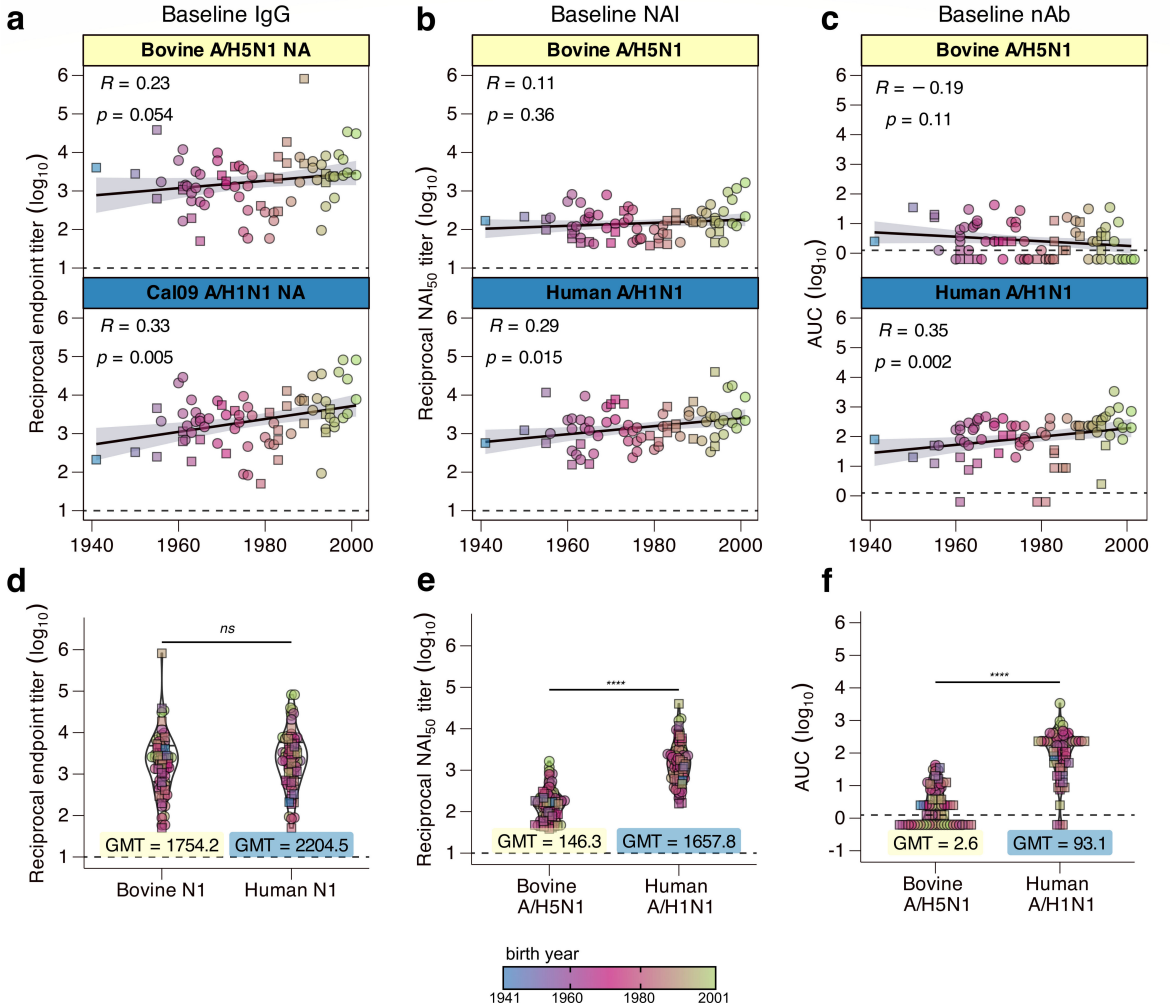
Baseline cross-reactive responses against A/H5N1 (*n* = 73 total at baseline timepoint; squares represent participants in the infection cohort, and circles represent participants in the vaccine cohort). Baseline blood serum was used to investigate baseline responses against A/H1N1 NA and A/H5N1 NA. (**a, d**) Enzyme-linked immunosorbent assay binding IgG against the bovine A/H5N1 NA (top panel, yellow label) and against a representative human seasonal A/H1N1 NA from A/California/04/2009 (bottom panel, blue label), plotted by (**a**) birth year and (**d**) antigen. (**b, e**) Enzyme-linked lectin assay NAI responses at baseline against an A/H5N1 bovine recombinant virus in a live attenuated influenza vaccine (LAIV) backbone (top panel, yellow label) and against a contemporary A/H1N1 seasonal virus (bottom panel, blue label), plotted by (**b**) birth year and (**e**) virus. (**c, f**) nAb titers at baseline against an A/H5N1 bovine recombinant virus in an LAIV backbone (top panel, yellow label) and against a contemporary A/H1N1 seasonal virus (bottom panel, blue label), plotted by (**c**) birth year and (**f**) virus. (**a–c**) Spearman correlation coefficient and significance are indicated in the upper left-hand corner of each panel. (**d–f**) Box-and-violin plots of the data shown in a–c with GMTs indicated for each (**d**) antigen or (**e, f**) virus. (**a–f**) Dotted lines indicate the lower limit of detection for the specified assay. (**a–c**) Line of best fit is indicated by the black line, with the standard error indicated by the shaded area in each panel. (**d–f**) Significance shown was generated via nonparametric one-way ANOVA with Dunn’s post-test. *ns* = not significant; **** = P < 0.0001.

To capture the range of baseline cross-reactivity across both cohorts, serum samples at the time of enrollment for the vaccine cohort and at the time of patient testing for the infection cohort were used to determine total binding IgG, NAI antibody, and nAb titers (Fig. 1). Baseline IgG binding titers were highly comparable against the bovine N1 NA and the NA from A/California/04/2009 (Cal09), which represents a human seasonal A/H1N1 pdm09-lineage NA. All participants had detectable binding IgG against the bovine and Cal09 NA at baseline, with geometric mean titers (GMTs) of 1,754.17 and 2,204.47, respectively (Fig. 1a). Although anti-Cal09 NA-binding titers were significantly correlated with birth year, this trend was less clear for anti-bovine N1 NA IgG (Fig. 1a). Baseline binding titers against both Cal09 NA and bovine A/H5N1 NA were significantly higher than baseline binding titers to bovine H5 HA, with a GMT of 364.88 (Fig. S2a). This suggests that pre-existing antibody titers against bovine N1 NA are greater than those against bovine H5 HA.

Using a recombinant bovine A/H5N1 virus whose multibasic cleavage site was deleted (∆MBS) and expressed in the genetic background of a live attenuated influenza vaccine virus (LAIV), hereafter referred to as bovine A/H5N1-LAIV, NAI titers were evaluated using an enzyme-linked lectin assay (ELLA). Unlike binding IgG, baseline NAI responses against the bovine N1 NA were markedly lower than those against a human seasonal N1 NA (Fig. 1b).

Bovine A/H5N1-LAIV nAb responses were significantly lower than nAb responses against human A/H1N1 (Fig. 1c), with 46% of subjects (24 of 50 vaccinees and 10 of 23 infected participants) having no detectable nAb titers at baseline (Fig. 1c). We detected a strong correlation between baseline H5-binding IgG and both participant birth year (Fig. S2a) and bovine A/H5N1 nAb titers (Fig. S2c). Conversely, only 0.04% of participants (3 of the 23 infected participants and none of the 50 vaccinees) had detectable nAb titers against A/H1N1 at baseline (Fig. 1c), which suggests that the detected H5-binding IgG were likely non-neutralizing in function. Taken altogether, pre-existing immunity at the population level to bovine A/H5N1 HPAI preferentially targets N1 NA rather than H5 HA.

### Seasonal vaccination does not boost cross-reactive bovine A/H5N1 responses

**Fig 2:**
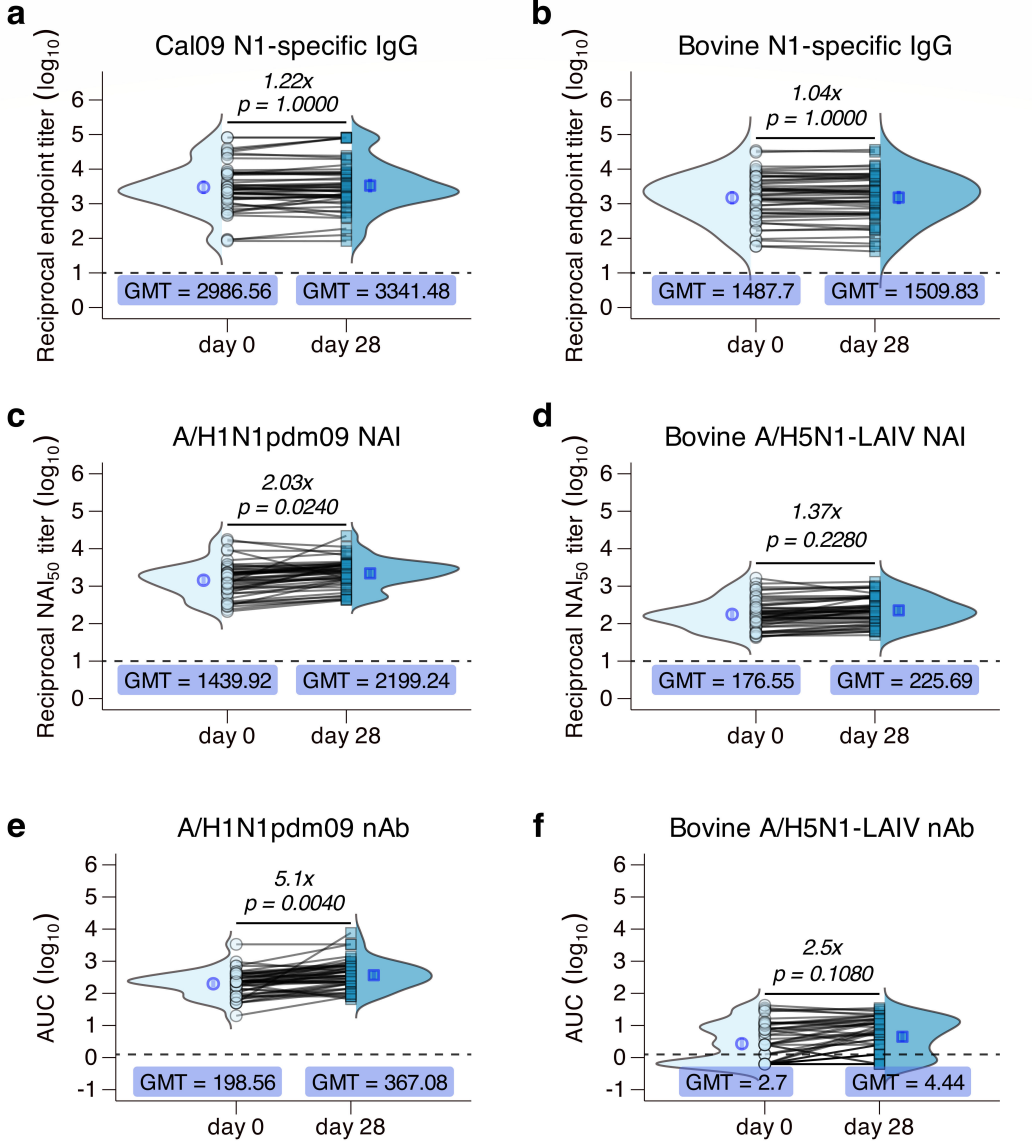
Vaccination does not induce cross-reactive NA-specific responses (*n* = 50). Seasonal vaccine recipients were enrolled on the date of seasonal influenza vaccine receipt, and 25 patients were selected at random per sex, stratified by age group. Serum was taken at the time of enrollment (indicated as “day 0,” light blue circles) and approximately 28 days later (indicated as “day 28,” turquoise squares), and subsequently used to evaluate A/H1N1 NA and A/H5N1 NA (**a, b**) total binding IgG via enzyme-linked immunosorbent assay, and (**c, d**) NA activity-inhibiting responses via ELLA, and (**e, f**) neutralizing antibody responses against an A/H1N1-like virus and bovine A/H5N1-LAIV virus. Dotted lines represent the assay lower limit of detection. Each symbol represents the arithmetic mean of two biological replicates for each sample. Lines between symbols indicate paired baseline and convalescent samples for a single patient. Arithmetic average fold change values are indicated for each panel, and *P*-values were generated by paired Wilcoxon signed-rank test. Blue open symbols and error bars represent the GMT and geometric 95% confidence interval, respectively. Ridge plots represent the total distribution of all data points.

Seasonal vaccines are currently the only widely available prophylactic for seasonal influenza epidemics (21–24). To address whether seasonal influenza vaccination can induce cross-reactive antibody responses to bovine A/H5N1 NA, pre- and post-vaccination NA-specific binding and inhibiting antibodies, as well as neutralizing antibodies, were quantified. It has been shown that although seasonal vaccination minimally increases detectable NA-specific antibodies, it often induces robust nAbs against the strains included in the vaccine formulation, primarily targeting the immunodominant HA head (21, 22, 24, 24–26). Unsurprisingly, seasonal vaccination did not significantly increase binding IgG titers against either human N1 NA or bovine N1 NA (Fig. 2a and b). ELLA revealed that vaccination had a statistically significant yet modest increase in NAI antibodies to human N1 NA, but no significant increases in NAI antibodies against bovine N1 NA (Fig. 2c and d). It should be noted that higher NAI titers post-vaccination may be partly influenced by HA-specific responses (27). Nonetheless, vaccination did not appear to boost cross-reactive NAI titers, which is consistent with binding IgG responses (Fig. 2a through d).

Because seasonal vaccines are formulated specifically with the aim of inducing neutralizing antibodies against the HA glycoprotein, we investigated whether vaccine-induced homotypic neutralizing antibodies could cross-neutralize A/H5N1 viruses. While nAb titers against A/H1N1pdm09 increased significantly after vaccination, there was no corresponding increase in A/H5N1-LAIV nAb titers (Fig. 2e and f). At day 28 post-vaccination, 13 of the initial 24 vaccinees who were seronegative remained seronegative, and two vaccinees saw a reduction in nAb titers to below the limit of detection (LOD) (Fig. 2f). There was no significant change, nor a consistent trend, in baseline binding IgG against bovine H5 HA following seasonal vaccination, as measured via enzyme-linked immunosorbent assay (ELISA) (Fig. S3a). Altogether, the data indicate that current seasonal vaccine formulations do not induce significant levels of binding or functional antibodies that recognize bovine H5 HA or N1 NA proteins.

### Boosting of A/H5N1 cross-reactive antibodies by seasonal IAV infection

**Fig 3:**
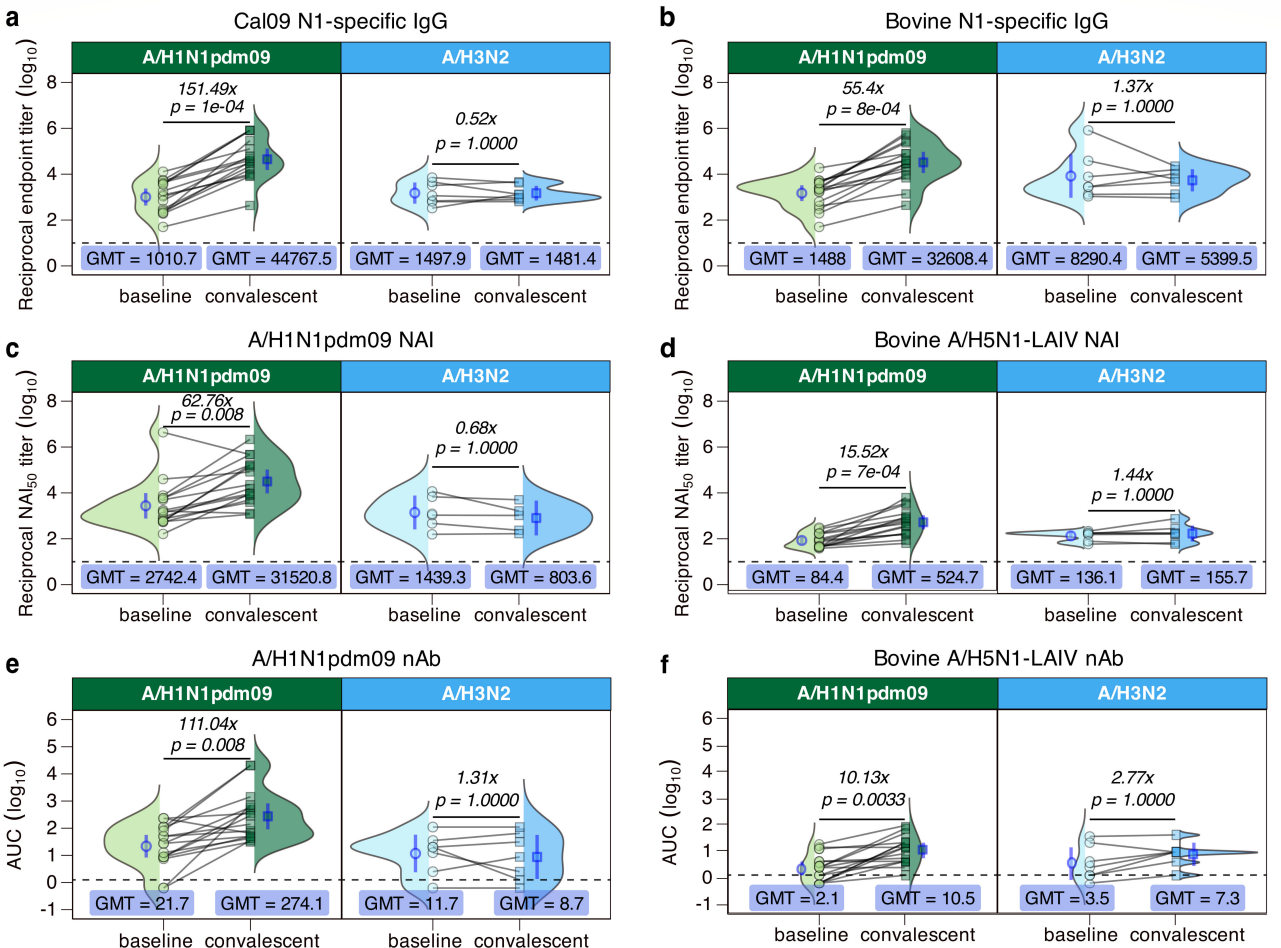
Seasonal A/H1N1 infection induces heterosubtypic NA-specific antibody responses to bovine A/H5N1 NA. (*n* = 23) Patients presenting to JHH Emergency Department with ILI and PCR-confirmed influenza A virus infection of either A/H1N1 subtype (*n* = 16, green) or A/H3N2 subtype (*n* = 7, light blue) were used to investigate NA-specific responses. Serum was taken at the time of enrollment (indicated as “baseline,” shown as circles) and approximately 28 days later (indicated as “convalescent,” shown as squares), and subsequently used to evaluate A/H1N1 NA and A/H5N1 NA (**a, b**) total binding IgG via ELISA and (**c, d**) NA activity-inhibiting responses via ELLA and (**e, f**) neutralizing antibody responses against an A/H1N1-like virus and bovine A/H5N1-LAIV virus. Gray lines represent the assay lower limit of detection. Dotted lines represent the assay lower limit of detection. Each symbol represents the arithmetic mean of two biological replicates. Lines between symbols indicate paired baseline and convalescent samples for a single patient. Arithmetic average fold change values are indicated for each panel, and *P*-values were generated by paired Wilcoxon signed-rank test. Blue symbols and error bars represent the GMT and geometric 95% CI, respectively. Ridge plots represent the total distribution of all data points.

Animal studies have suggested that infection with human seasonal A/H1N1 viruses can provide partial or complete protection against an A/H5N1 challenge (28–31). Unlike seasonal vaccination, IAV infection is known to reliably induce NA-specific antibody responses (18, 23, 27, 32, 33). NA-specific antibody responses were measured in our infection cohort (Fig. 3). For the 16 patients who had sequence-confirmed A/H1N1 infections, Cal09 NA- and bovine A/H5N1 NA-specific IgG were both substantially boosted by infection, albeit to different magnitudes (Fig. 3a and b), with a 151-fold increase in Cal09 N1 NA-binding antibodies and a 55-fold increase in bovine A/H5N1 NA-binding antibodies. For A/H3N2-infected patients, there was no consistent trend in binding IgG against either human or bovine N1 NA (Fig. 3a). These data suggest that A/H1N1, but not A/H3N2, infection boosts bovine N1 NA-specific IgG responses.

The trends we observed in NA-binding IgG for A/H1N1-infected patients were recapitulated in NAI titers (Fig. 3c and d). All but 1 of 16 patients increased in A/H1N1 NAI antibodies, and the fold increase was 62× (Fig. 3c). All 16 patients had a significant increase in A/H5N1-LAIV NAI titers post-infection but with a lower fold increase of 15.52× (Fig. 3d). A/H3N2-infected individuals did not show significant increases in NAI titers to human or bovine H1N1 viruses (Fig. 3c and d). There were also significant increases in nAb titers to both A/H1N1pdm09 and bovine A/H5N1 viruses after A/H1N1 infection but not after A/H3N2 infection (Fig. 3e and f). In addition to the increase in NAI and nAb responses to bovine A/H5N1-LAIV in the A/H1N1-infected group, H5 HA-binding IgG titers significantly increased post-infection by an average 10.34-fold (Fig. S3b). Altogether, A/H1N1-infected patients were the only group to see an increase in both HA- and NA-specific cross-reactive antibodies against bovine A/H5N1 HA and NA proteins.

### Investigating the contribution of heterotypic N2-specific antibodies to total A/H5N1 NA-binding antibody

Given the relatively high degree of conservation with respect to structural and functional domains of the NA protein, we determined whether heterotypic antibody to the N2 NA may contribute to the detectable binding IgG at baseline. Pan-NA mAbs often target domains of NA that are essential for either enzymatic function or overall structure of the NA tetramer and therefore conserved across IAV subtypes (34–37). To address the overall contribution of N2-specific binding IgG to the observed baseline bovine NA IgG titers, serum from a representative subset of our vaccine cohort—balanced by age and sex—was depleted of N2-binding IgG (Fig. S4a). We used a representative A/H3N2 recombinant NA derived from a locally circulating isolate in Baltimore, MD, for all N2-serum depletions. On average, sera showed binding IgG titers that were threefold higher than N2-depleted sera against bovine NA (Fig. S4a), indicating N2 NA cross-reactive antibodies make up a small portion of the cross-reactive bovine NA antibodies. We confirmed that our N2-depleted sera had little to no detectable binding against the same A/H3N2 NA (Fig. S4b), further suggesting that pre-existing cross-reactive antibodies may be made up of both heterosubtypic NA-targeting antibodies as well as heterotypic NA-targeting antibodies. These findings suggest that heterotypic antibodies, namely those that bind N2 NA, may partially contribute to observed baseline bovine NA antibody responses and substantiate further investigation of cross-group neuraminidase antibodies.

## DISCUSSION

A key component of risk assessment for A/H5N1 HPAI is a comprehensive understanding of pre-existing immunity at the population level (38–40). In the present work, we illuminate the distinct roles of seasonal human IAV infection and vaccination in shaping baseline cross-reactive antibody repertoire to the NA of A/H5N1 viruses. The majority of candidate pandemic HPAI vaccines against A/H5N1 are centered around the HA glycoprotein (17, 41–45). Similarly, available reports detailing the pre-existing antibody repertoire against A/H5N1 are focused on the detection of cross-reactive neutralizing antibodies, which target HA (45–49). Antibodies against NA are often not neutralizing in function (18, 50–54). While H5 HA is structurally and antigenically distinct from human seasonal H1 HA, they are part of the same phylogenetic group—group 1—and share some identity in sequence, particularly within the more conserved HA stem (49, 55–57). Given the immunodominance of HA during both vaccination and infection, there is a possibility that H5-specific responses at baseline are contributing to the detectable NAI response to bovine A/H5N1-LAIV (27) and might also be neutralizing. Other studies have detected low nAb responses against A/H5N1 clades (19, 45, 47, 48). Nonetheless, it has long been established that NA-targeting antibodies are an important mediator of protective immunity against IAV, albeit these responses are often not captured in traditional serological assessments like neutralizing antibody assays and hemagglutination inhibition assays (18, 32, 33, 52, 58–61). Therefore, N1 NA acting as a shared target between human seasonal A/H1N1 viruses and A/H5N1 HPAI likely allows for the detectable cross-reactive NA-targeting responses we and others report (17, 19, 45, 48).

Our work describes pre-existing immunity in a population of healthcare workers from JHH (Fig. 2). Understanding the level of baseline immunity against A/H5N1 in this population is essential when considering the pandemic potential of A/H5N1, and who is most at risk of occupational or incidental exposures—with healthcare workers being at high risk, only second to dairy and poultry farmers who have direct contact with infected animals. Because vaccination is mandated each season for JHH employees, this cohort allows us to investigate the vaccine-induced antibody repertoire as it pertains to cross-reactive bovine A/H5N1 HPAI responses without sacrificing the heterogeneity present at the population level, which is often lost in animal studies (29, 46, 62, 63). Among healthcare workers, baseline binding IgG against Cal09 NA and against bovine A/H5N1 NA were similar to those among our infection cohort at baseline. When considered with our correlation analyses, binding IgG against both Cal09 NA and bovine A/H5N1 NA appeared to be positively correlated with birth year, although this was not statistically significant for bovine A/H5N1 NA (Fig. 1a). This trend was consistent for NAI titers, but we saw a slight negative correlation for nAb responses to bovine A/H5N1-LAIV (Fig. 1b and c). When considering A/H5N1 HA responses, the slight negative correlation with birth year and nAb titer was more exaggerated between birth year and bovine A/H5N1 HA-binding IgG (Fig. S2a). These data highlight important factors and considerations that underlie observed heterogeneity in pre-existing immunity to A/H5N1, such as primary influenza exposures and original antigenic sin (64).

For seasonal influenza infections, A/H1N1 infection was clearly correlated with the induction of cross-reactive nAb and bovine N1 NA antibodies (Fig. 3), which was absent in A/H3N2-infected individuals. At least 25% of our infection cohort reported no receipt of a seasonal influenza vaccine in any of the past five seasons, and 11 of 23 had not received the seasonal vaccine during the season that they were infected (Table 2). This suggests that any differences in baseline NA-specific IgG are unlikely a consequence of vaccine-induced immune responses. However, it is worth noting that the size of our A/H3N2-infected cohort is limited, as the 2023–2024 NH influenza season was dominated by A/H1N1 viruses (65). Thus, we plan to extend this work to the most recent (e.g., the 2024–2025) NH season and to future influenza seasons to increase the sizes of both our A/H1N1- and A/H3N2-infected cohorts. Furthermore, while we detect increases in bovine H5 HA (Fig. S3c) and N1 NA-binding (Fig. 3b) antibody responses after infection, these levels are much lower than those targeting human seasonal HA or NA proteins. Since there are no established correlates of protection for A/H5N1 infection in the human population, we cannot make any conclusions about how these cross-reactive antibodies against bovine A/H5N1 might modulate infection or disease severity. Nonetheless, our data merit additional study for defining immune correlates of protection for both HA- and NA-targeting responses.

Seasonal vaccination did not change H5-binding IgG titers or bovine A/H5N1-LAIV nAb titers, which is expected due to the immunodominance of the globular HA head in vaccine-induced antibody responses (21, 22, 25, 66, 67). This corroborates previously published data, which indicate that despite no known exposures, cross-reactive antibodies to H5 HA are detectable, albeit minimal (19, 45, 48). While NA has been increasingly considered as a potential immunogen for universal influenza vaccines, our current understanding of how NA-specific responses can mediate immunity is limited. Current practices for seasonal vaccine development and production do not include quantifying NA content (21, 66), despite its role as the common denominator between human influenza viruses and several zoonotic influenza viruses (such as, but not limited to, swine A/H1N1, swine A/H1N2, swine A/H3N2, canine A/H3N2, avian A/H1N1, avian A/H2N2, avian A/H3N2, avian A/H5N1, avian A/H9N2, etc.). We show the relevance of A/H1N1 infection, as only A/H1N1 infection was capable of significantly boosting binding, neutralizing, and NA-inhibiting antibodies against bovine A/H5N1 (Fig. 3). Relative to HA, NA is a slower-moving antigenic target and has lower mutational plasticity (21, 61, 66). It must maintain its vital enzymatic function for productive influenza virus infections, which provides a common target across not only IAVs but also extends to influenza B viruses. Despite the discovery of several pan-NA mAbs that have shown protection in lethal challenge models against a panel of human and zoonotic IAVs (18, 32, 34, 35, 37, 68–70), NA remains comparatively understudied and overlooked as a viral and vaccine antigen for broadly protective immunity (58, 60, 61, 71). While there have been far fewer clinical trials investigating NA-based vaccine immunogenicity and efficacy, phase I studies of vaccines that include NA as an immunogen highlight its potential for induction of robust homotypic and heterotypic antibodies that are durable (72, 73). Few NA-only vaccines have been evaluated in humans, although many have shown protection in lethal challenge models (16, 33, 34, 36, 59, 70, 74–76). The lack of human immunogenicity data, efficacy data, and transmission data for NA vaccine candidates opposes the push toward including NA in current vaccines. Until sufficient human clinical trials have been conducted to support non-inferiority relative to current seasonal influenza vaccines against pandemic strains, the total utility of including NA or switching to NA-only vaccine designs remains uncertain.

We show evidence that supports changing current seasonal vaccine formulations to either include greater NA content or to manipulate immunogen design to increase immunofocusing toward immunosubdominant domains of IAV HA and NA (25, 66, 77, 78). The work presented here reiterates the importance of NA as a conserved antigen between human seasonal viruses and A/H5N1 HPAIs and underscores the need for investigation of NA-mediated antibody responses and their role in protective immunity. NA-centered vaccine design would enable robust boosting of cross-reactive N1 antibodies and may serve as a more feasible approach to increasing population-level pre-existing antibodies to A/H5N1 compared to HA-focused vaccines.

## MATERIALS AND METHODS

### Human subjects enrollment, sampling, and data collection

Serum used in this study was obtained from healthcare workers recruited during the annual Johns Hopkins Hospital employee influenza vaccination campaign in the Fall of 2024 by the Johns Hopkins Centers for Influenza Research and Response. Pre- (immediately prior to vaccination) and post (~28 day) vaccination human serum was collected from subjects, who provided written informed consent prior to participation. Patients were enrolled at the JHMI Department of Emergency Medicine or on inpatient floors. Symptomatic patients in the emergency department were screened and tested for influenza from triage by clinical providers using a validated clinical decision guideline tool. Serum was collected at the time of presentation and approximately 28 days later.

### Viruses

Virus isolates used in this study are as follows: A/Victoria/4897/2022 (A/H1N1 pandemic09-like, 2024–2025 NH vaccine virus; courtesy of John Steel, CDC). Recombinant viruses used in this study include A/Baltimore/R0675/2019 (A/H1N1 pandemic09-like, GISAID accession no. EPI_ISL_17617226) and an LAIV-like virus expressing the HA and NA segments of bovine A/H5N1 (GISAID accession no. EPI_ISL_19014384), which were used for ELLA quantification of NAI antibody and neutralizing titer 50% (NT_50_) assay for detection of nAb responses. A/Baltimore/R0675/2019 was generated by a 12-plasmid reverse genetics system for the generation of influenza A viruses (79). Transfection of co-cultured Madin-Darby Canine Kidney (MDCK)-SIAT1 and HEK-293T cells with plasmids encoding each of eight segments belonging to A/Baltimore/R0675/2019 and four helper plasmids encoding the polymerase complex of influenza viruses was conducted as previously described (80, 81). Recombinant viruses expressing the HA and NA segments of A/Bovine/Texas/24-029328-01/2024 (GISAID accession no. EPI_ISL_19014384) were generated as follows: the multibasic cleavage site in HA was mutated to delete the RRKR motif (amino acid positions 342 to 346) to make the virus dependent upon exogenous trypsin (28). HA and NA segments from A/Bovine/Texas/24-029328-01/2024 were cloned into an eight-plasmid expression system with bidirectional promoters (courtesy of Dr. Seema Lakdawala, Emory University, and Dr. Valerie Le Sage, University of Pittsburgh) (80, 81). The six remaining gene segments encoding the internal genes of A/Ann Arbor/6/1960 (H2N2) (GISAID accession no. EPI_ISL_130415), a cold-adapted virus (live attenuated, indicated as LAIV), cloned into the 12-plasmid reverse genetics system, were co-transfected with the bovine-derived A/H5N1 HA and NA described above, with co-cultured MDCKI and HEK-293T cells, as previously described (79–83).

### Cell lines and maintenance

MDCK derivatives, MDCKI and MDCK-SIAT1 (courtesy of Dr. Scott Hensley, University of Pennsylvania), were maintained in cell culture in complete media—hereafter referred to as CM—consisting of Dulbecco’s modified Eagle medium (DMEM; Gibco) supplemented with 10% fetal bovine serum (FBS; Gibco), 100 U/mL penicillin/streptomycin (Life Technologies), and 2 mM GlutaMAX (Gibco). Cells were passaged by washing 2× with PBS (1×; Life Technologies), followed by treatment with trypsin-EDTA (0.5%) (Gibco) and incubation at 37°C for up to 15 minutes, at which point cells had detached. Trypsin was quenched by the addition of an equal volume of CM. Cells were either subsequently passaged or were plated to be used for virus propagation, virus quantification by tissue culture infectious dose 50% (TCID_50_) assay, and NT_50_ assay.

### Recombinant neuraminidase HA and NA protein

Recombinant bovine A/H5N1 HA (derived from the A/dairy cow/Texas/24-008749-002-v/2024 strain) was obtained from Sino Biological (cat no. 41036-V08H). Protein was reconstituted per the manufacturer’s instructions to 1 mg/mL and stored at −80°C until use. NA sequences were designed as previously described (84, 85). Briefly, the cytoplasmic, transmembrane, and stalk domains of wild-type NA were replaced with an N-terminal signal sequence, 6×His tag, a tetramerization domain from the human vasodilator-stimulated phosphoprotein, a thrombin cleavage site, and a linker sequence followed by the NA sequence (86). NA constructs were expressed in Expi293F cells and purified by Ni-NTA chromatography as previously described (87).

### ELISA for quantification of antigen-specific IgG

Antigen-specific IgG was quantified by ELISA as previously described (88, 89). Protein was diluted in 1× PBS (Life Technologies) to 1 µg/mL for all neuraminidase constructs and to 0.5 µg/mL for bovine H5 hemagglutinin (Sino Biological), added to Nunc MaxiSorp 96-well plates (Thermo Fisher), and incubated at 4°C for 16 hours. Plates were washed with 1× PBS supplemented with 0.1% Tween 20 (Sigma), designated as PBST. Heat-inactivated serum samples were serially diluted fourfold, eight times in blocking buffer, which was composed of PBST + 5% skim milk (Thermo Fisher). Diluted sera were added to plates in duplicate and incubated at room temperature for 1 hour. Goat anti-human IgG (gamma chain) cross-adsorbed horseradish peroxidase (HRP) conjugate was used as a secondary antibody. TMB substrate (Thermo Fisher) was added to all wells and incubated in the dark for 18–20 minutes. The reaction was stopped by the addition of 0.16 M sulfuric acid, and plates were read at OD_450_ and OD_650_ with background subtraction. Reciprocal endpoint titers were determined as the serum dilution that yielded signal four times that of secondary antibody alone.

### ELLA for quantification of NAI antibody responses

ELLA was used to quantify neuraminidase activity-inhibiting antibodies. In brief, Nunc 96-well Immulon 4 HBX plates (Thermo Fisher) were coated with fetuin from FBS (Sigma) diluted in 1× PBS to 2.5 µg/well. Plates were sealed and stored at 4°C for 12 to 16 hours. Sera were serially diluted fourfold, eight times in assay buffer, which consisted of 0.2% Tween 20 (Sigma), 1% BSA (Sigma), 0.1 mg/mL MgCl_2_, and 0.2 mg/mL CaCl_2_, diluted in 1× PBS. A/H1N1 or A/H5N1-LAIV was appropriately diluted in assay buffer and added to serially diluted serum and incubated at 37°C for 1 hour. During the 1 hour incubation, fetuin-coated plates were washed 3× with PBST. Sera/virus mixtures were added to washed plates in duplicate, and each plate contained eight wells with only assay buffer (to represent no NA activity, or 100% NAI) and an additional eight wells with virus alone and no antibody (to represent full NA activity, or 0% NAI). Plates were then sealed and incubated for 16 to 18 hours at 37°C with 5% CO_2_. Plates were carefully washed with PBST and incubated at room temperature for 2 hours with HRP-conjugated lectin from *Arachis hypogaea*, also referred to as HRP-conjugated peanut agglutinin (Sigma). Plates were washed a final time and then reacted with SigmaFast OPD substrate (Sigma) away from direct light for approximately 10 minutes. Reactions were stopped by the addition of an equivalent volume of 1 N H_2_SO_4_, and OD was read at 490 nm to determine relative NAI.

### NT_50_ assay for quantification of nAb responses

NT_50_ assays were conducted as previously reported (90). Human sera obtained as described above were treated with lyophilized receptor-destroying enzyme II (RDE; Hardy Diagnostics) per the manufacturer’s instructions. For A/H1N1 and A/H5N1-LAIV viruses, MDCK-SIAT1 cells (courtesy of Dr. Scott Hensley) and MDCKI cells were seeded in CM in 96-well plates (Celltreat) 2 days prior to infection. RDE-treated sera were serially diluted in DMEM supplemented with 100 U/mL penicillin/streptomycin (Life Technologies), 2 mM GlutaMAX (Gibco), 0.3% bovine serum albumin (Sigma), and 1 or 5 µg/mL of N-acetylated trypsin (Sigma) for assays conducted on MDCK-SIAT1 cells or on MDCKI cells, respectively. One hundred TCID_50_ was added to each well of serially diluted serum and incubated at 33°C with 5% CO_2_ for 1 hour prior to infecting cells. The serum and virus mixture was added to cells in quadruplicate and incubated at 33°C 5% CO_2_ for 24 hours. After 24 hours, all plates were washed twice with 1× PBS supplemented with CaCl_2_ and MgCl_2_, and the media were replaced before plates were returned to 33°C with 5% CO_2_. One hundred twenty hours later, plates were fixed with 10% neutral buffered formalin (Leica) and stained with naphthol blue-black for subsequent interpretation. Area under the curve was calculated using GraphPad Prism 10.4.2. Curves were generated by entering the fraction of all four wells per sample protected at each dilution factor. The LOD was determined to be the smallest possible AUC value generated, e.g., when only one of four wells was protected at the first dilution in the full dilution series. Any samples that had no detectable neutralizing titer were set to be equal to one-half of the assay LOD for use in calculating GMTs.

### Serum depletion of antigen-specific antibody

Baseline human sera was depleted of antigen-specific antibody by coupling His-tagged recombinant A/H3N2 NA tetramers from A/Baltimore/R0145/2017(H3N2) (GenBank: MH637451) to magnetic His Dynabeads (Invitrogen; Thermo Fisher) as previously described (89). Briefly, 5 µL of heat-inactivated sera was incubated with 195 µL of N2Baltimore2017 diluted in PBS, for a total of 200 µL, at room temperature with constant agitation for 1 hour. Washed magnetic beads were resuspended in 50 µL of PBS and incubated at room temperature with constant agitation for approximately 30 minutes. Tubes containing the above three components were placed on a magnetic strip (Invitrogen; Thermo Fisher) and incubated until all beads had precipitated out of solution. The supernatant was collected and subjected to ELISA to confirm the successful elimination of all N2Baltimore2017-specific IgG and to quantify remaining bovine A/H5N1 NA-binding IgG.

### Data analysis

All statistical analyses shown were calculated in RStudio (version 2025.05.1+513) using either base R or the rstatix packages (91). Appropriate tests were run as indicated in figure legends. Area under the curve and non-linear regressions were performed either in GraphPad Prism 10.4.2 or in RStudio. All graphs were generated in RStudio.

## Data Availability

All data are available through the Johns Hopkins Data Repository (https://doi.org/10.7281/T1/KHIQAT).

## SUPPLEMENTAL MATERIAL

Figure S1 mbio.02145-25-s0001.TIFF Study design schema.

Figure S2 mbio.02145-25-s0002.TIFF Baseline antibody responses to bovine A/H5N1 HA.

Figure S3 mbio.02145-25-s0003.TIFF Vaccine- and infection-induced bovine A/H5N1 HA responses.

Figure S4 mbio.02145-25-s0004.TIFF Serum depletion of N2-specific antibody decreases bovine N1 binding IgG.

Legends mbio.02145-25-s0005.docx Legends for all supplemental figures.

ASM does not own the copyrights to Supplemental Material that may be linked to, or accessed through, an article. The authors have granted ASM a non-exclusive, world-wide license to publish the Supplemental Material files. Please contact the corresponding author directly for reuse.
