# Supplementary figures and images for: Low levels of influenza H5N1 HA and NA antibodies in the human population are boosted by seasonal H1N1 infection but not by H3N2 infection or influenza vaccination
Source: mBio. 2025 Oct 31;16(12):e02145-25. doi: 10.1128/mbio.02145-25 (PMC12691596; doi:10.1128/mbio.02145-25)

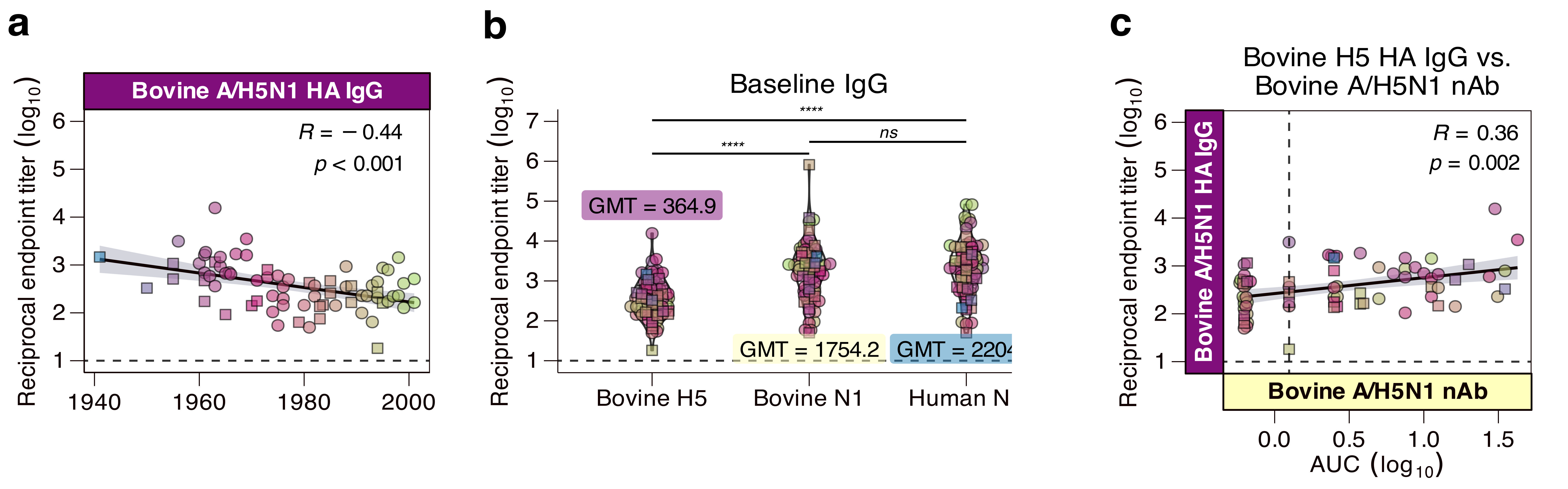

Supplement: Figure S2 — Baseline antibody responses to bovine A/H5N1 HA. [file mbio.02145-25-s0002.tiff]

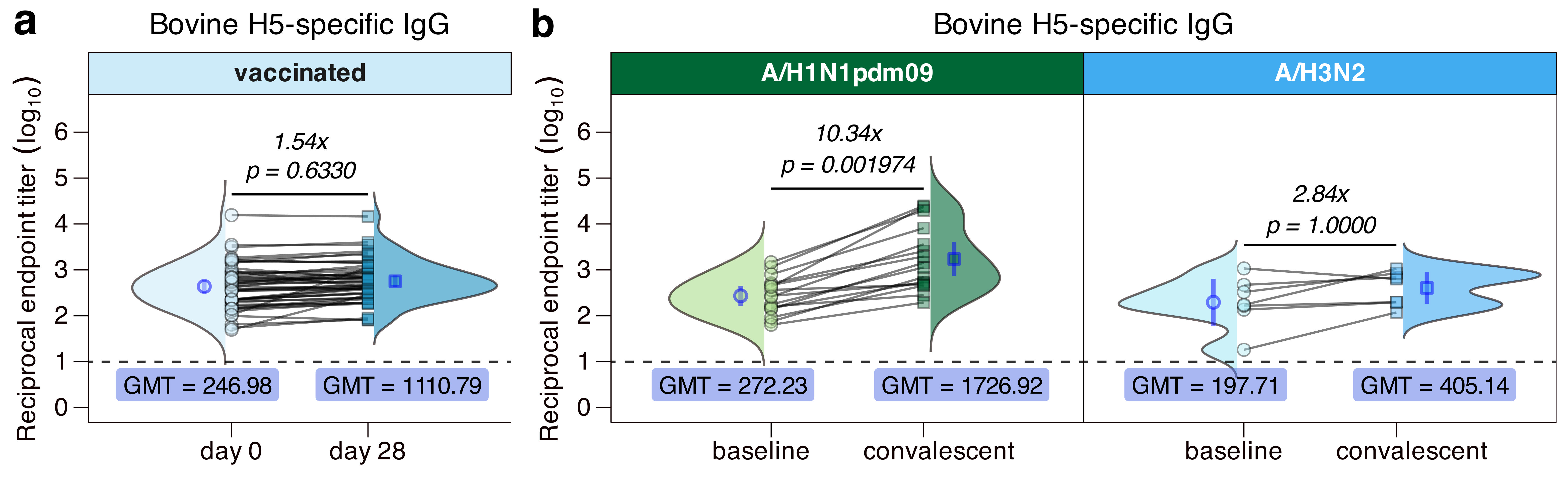

Supplement: Figure S3 — Vaccine- and infection-induced bovine A/H5N1 HA responses. [file mbio.02145-25-s0003.tiff]

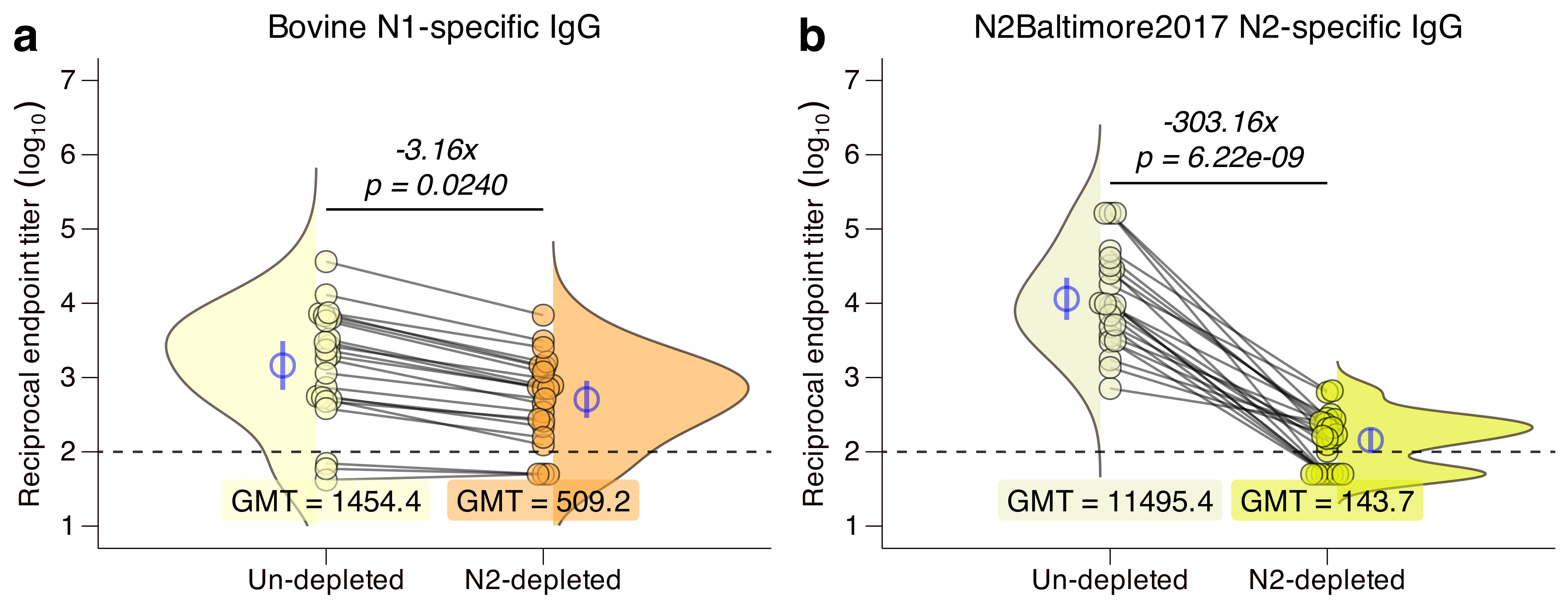

Supplement: Figure S4 — Serum depletion of N2-specific antibody decreases bovine N1 binding IgG. [file mbio.02145-25-s0004.tiff]
