# Supplementary material for: Low levels of influenza H5N1 HA and NA antibodies in the human population are boosted by seasonal H1N1 infection but not by H3N2 infection or influenza vaccination
Source: mBio. 2025 Oct 31;16(12):e02145-25. doi: 10.1128/mbio.02145-25 (PMC12691596; doi:10.1128/mbio.02145-25)
Supplement: Legends — for all supplemental figures. [file mbio.02145-25-s0005.docx]

**Supplemental Figure 1. Study design schema.** (**top**) vaccine cohort study timeline and composition. (**bottom**) infection cohort study timeline and composition. Made with Biorender.

**Supplemental Figure 2. Baseline antibody responses to bovine A/H5N1 hemagglutinin (HA).** Baseline binding IgG (**a-b**) against soluble bovine A/H5N1 HA was measured for all samples (n = 73) at the time of enrollment by standard ELISA, plotted by birth year and (**b**) by antigen. Squares represent samples from the infection cohort (n = 23) and circles represent samples from the vaccine cohort (n = 50). (**a**) Spearman coefficient was calculated between baseline binding IgG to A/H5N1 HA and birth year. (**b**) Anti-bovine A/H5N1 HA IgG at baseline was compared to bovine A/H5N1 NA and Cal09 NA binding responses. (**c**) Spearman correlation analyses for binding IgG responses against (**c)** bovine A/H5N1 nAb responses. (**b**) non-parametric one-way ANOVA with Dunn’s post-test shows significantly lower baseline binding IgG titers against bovine A/H5N1 HA than against either NA; ***** = p < 0.0001*, *ns = not significant*. (**b**) geometric mean titer (GMT) is indicated for each group, with H5 binding IgG GMT indicated in purple. (**a, c**) Spearman correlation coefficient and significance are indicated in the upper lefthand corner of each panel. (**a-c**) Dotted lines indicate the lower limit of detection for the specified assay. Line of best fit is indicated by the black line, with the standard error indicated by the shaded area in each panel.

**Supplemental Figure 3. Vaccine- and infection-induced bovine A/H5N1 HA responses.** (**a**) serum obtained from vaccine recipients at days 0 (circles) and 28 (squares) post-vaccination were subject to standard binding ELISA to quantify serum IgG against the bovine H5 HA. (**b**) The same quantification of bovine H5 HA-binding IgG was performed on serum from patients infected with circulating (left panel, green) A/H1N1pdm09 or (right panel, blue) A/H3N2 at the time of admittance to the JHH ED (indicated as “baseline,” represented by circles) and approximately 4 weeks later (indicated as “convalescent,” represented by squares). (**a-b**) Dotted lines represent the assay lower limit of detection. Each symbol represents the arithmetic mean of two biological replicates for each sample. Lines between symbols indicate paired baseline and convalescent samples for a single patient. Arithmetic average fold-change values are indicated for each panel, and p-values were generated by paired Wilcoxon signed-rank test. Blue symbols and error bars represent the geometric mean titer (GMT) and geometric 95% CI, respectively. Ridge plots represent the total distribution of all datapoints.

**Supplemental Figure 4. Serum depletion of N2-specific antibody decreases bovine N1 binding IgG.** (n = 23) Serum obtained from the vaccine cohort, stratified by age and sex, at the time of vaccination was depleted of N2-binding antibodies, and subject to ELISA to quantify (**a**) bovine A/H5N1 NA and (**b**) A/H3N2 NA binding titers. Dotted lines represent the assay lower limit of detection. (**a-b**) Each symbol represents the arithmetic mean of two biological replicates for each sample. Lines between symbols indicate paired baseline and convalescent samples for a single patient. Arithmetic average fold-change values are indicated for each panel, and p-values were generated by paired Wilcoxon signed-rank test. Blue symbols and error bars represent the geometric mean titer (GMT) and geometric 95% CI, respectively. Ridge plots represent the total distribution of all datapoints.
